# Intraspecific differences in molecular stress responses and coral pathobiome contribute to mortality under bacterial challenge in *Acropora millepora*

**DOI:** 10.1038/s41598-017-02685-1

**Published:** 2017-06-01

**Authors:** Rachel M. Wright, Carly D. Kenkel, Carly E. Dunn, Erin N. Shilling, Line K. Bay, Mikhail V. Matz

**Affiliations:** 1 Department of Integrative Biology, University of Texas at Austin, 205 W. 24th Street C0990, Austin, TX 78712 USA; 2 Australian Institute of Marine Science, PMB No. 3, Townsville MC, Queensland 4810 Australia

## Abstract

Disease causes significant coral mortality worldwide; however, factors responsible for intraspecific variation in disease resistance remain unclear. We exposed fragments of eight *Acropora millepora* colonies (genotypes) to putatively pathogenic bacteria (*Vibrio spp*.). Genotypes varied from zero to >90% mortality, with bacterial challenge increasing average mortality rates 4–6 fold and shifting the microbiome in favor of stress-associated taxa. Constitutive immunity and subsequent immune and transcriptomic responses to the challenge were more prominent in high-mortality individuals, whereas low-mortality corals remained largely unaffected and maintained expression signatures of a healthier condition (*i.e*., did not launch a large stress response). Our results suggest that lesions appeared due to changes in the coral pathobiome (multiple bacterial species associated with disease) and general health deterioration after the biotic disturbance, rather than the direct activity of any specific pathogen. If diseases in nature arise because of weaknesses in holobiont physiology, instead of the virulence of any single etiological agent, environmental stressors compromising coral condition might play a larger role in disease outbreaks than is currently thought. To facilitate the diagnosis of compromised individuals, we developed and independently cross-validated a biomarker assay to predict mortality based on genes whose expression in asymptomatic individuals coincides with mortality rates.

## Introduction

Global declines in coral cover are compounded by a variety of diseases^1, 2^, many of which are ambiguously defined by macroscopic characterizations of lesions^1–3^. Several bacterial species from the genus *Vibrio* have been implicated as etiological agents of some coral diseases^4–6^, but these bacteria may act merely as opportunistic pathogens exploiting compromised hosts^7^. The reported presence of pathogens on healthy colonies^8^ and diversity of bacterial species associated with diseased colonies^9, 10^ suggests that many instances of that coral disease cannot be attributed to a single pathogen. Instead, multiple bacteria appear to act opportunistically during coral disease events^11^. To describe these recurrent phenomena in coral disease biology, researchers have recently introduced the concept of the pathobiome^12^. Instead of a single etiological agent, the pathobiome consists of all microbiota that contribute to disease and describes interactive effects between microbiome members and environmental conditions^13^.

Host immune health is considered to be a major determinant of disease transmission dynamics^14, 15^. Corals, like all invertebrates, rely entirely on innate immunity for protection from invading pathogens. Features of innate immunity in corals include molecular pattern recognition^16^, secreted antimicrobial macromolecules^17^, cellular responses (*e.g*., phagocytosis)^18, 19^, and physical barriers (*e.g*., mucus)^20, 21^. Melanin deposits serve as another physical barrier against invading pathogens^22^. The melanin synthesis cascade is activated when pathogen recognition triggers cleavage of prophenoloxidase (PPO) to phenoloxidase (PO). Reactive oxygen species (ROS) produced during melanin synthesis contribute to its cytotoxic effects on pathogens, but also cause self-harm^23^ that must be countered by antioxidant enzymes such as catalase (CAT) and peroxidase (POX).

Field surveys of naturally occurring coral disease outbreaks show marked variability in mortality among conspecifics, despite the fact that neighboring colonies are exposed to the same environmental stressors and, presumably, the same potential pathogens^14, 24^. One possible explanation is that some corals resist disease by making greater contributions to constitutive or inducible immunity. Coral families that invest more in innate immunity (*e.g*., production of cytotoxic defenses) are less likely to suffer infectious disease outbreaks^25, 26^. One of the hardiest coral species in the Caribbean – *Porites astreoides* – is characterized by elevated immunity compared to species from other coral families^25^. However, no laboratory experiments have yet addressed the role of immunity or other molecular characteristics in driving variation in disease outcomes among conspecifics in any coral species.

In this study, we comprehensively examine coral host immune activity, genome-wide gene expression, *Symbiodinium* profiles, and coral-associated microbial communities in a bacterial challenge experiment to understand the physiological and molecular features underpinning intraspecific variation in mortality rate. Fragments of eight colonies of *Acropora millepora* from two locations on the Great Barrier Reef (GBR) were individually challenged in a full-factorial design with bacteria (*Vibrio owensii* and *V. diazotrophicus*) and mechanical abrasion (n = 3 per experimental group, Fig. S1). These fragments were monitored over a week of lesion development and progression. Unchallenged control samples were used to identify baseline correlations of constitutive physiological parameters with survivorship. Post-challenge, yet asymptomatic, samples demonstrated genotype-specific gene expression responses to the bacterial challenges.

## Results

### *A. millepora* genotypes show significant differences in mortality

**Figure 1:**
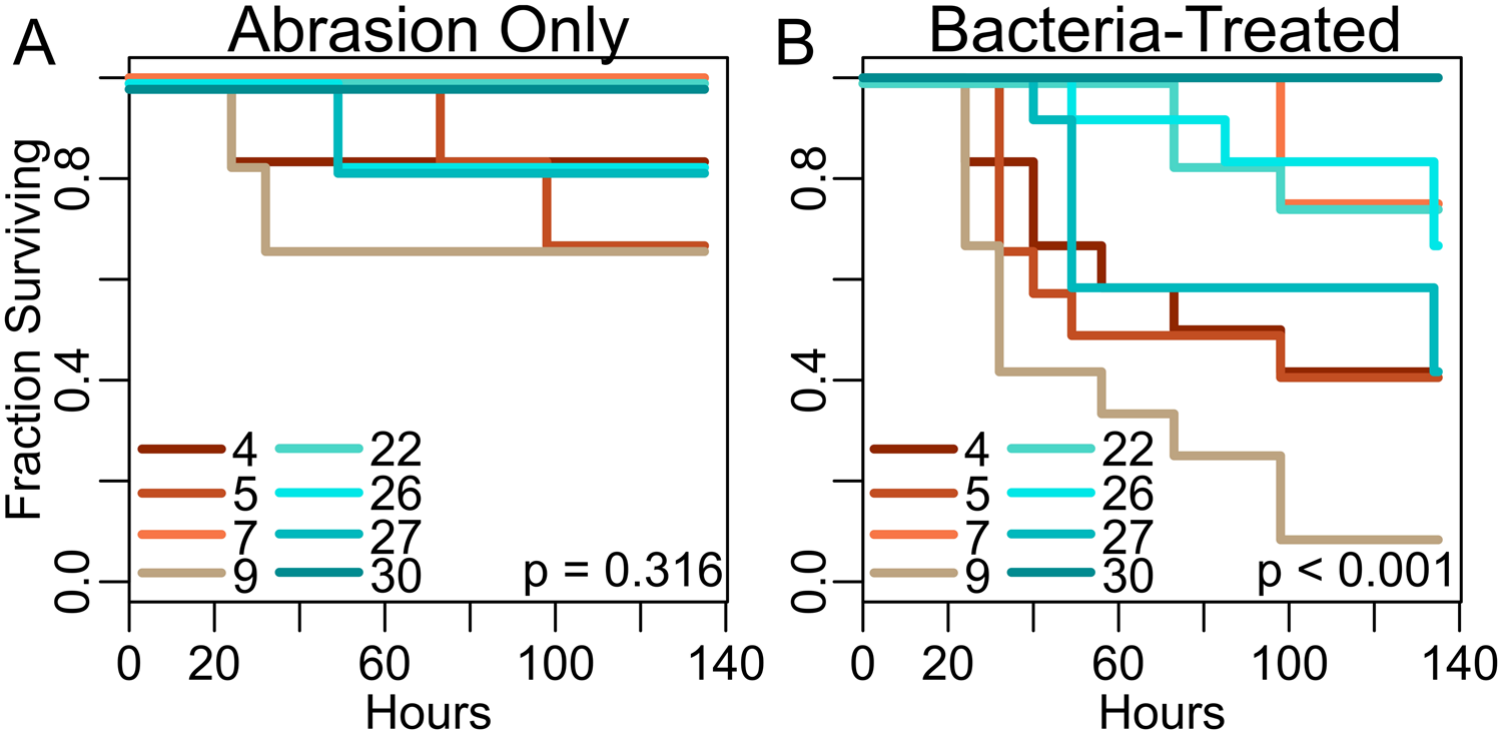
Differences in survival among *A. millepora* genotypes. (**A**) Survival of abraded fragments without bacterial challenge. (**B**) Survival of bacteria-treated fragments (abraded and non-abraded). Differential mortality among genotypes is only observed in response to the bacterial challenge. No mortality was observed among control fragments. P-values correspond to the effect of genotype in a Cox proportional hazards model. Genotypes 4, 5, 7, 9 are from Lizard Island, genotypes 22, 26, 27, 30 are from Wilkie Island.

No lesions formed under control conditions in any genotype, whereas some fragments died after abrasion (Fig. 1A) and many more died under bacterial or combined treatment (Fig. 1B). There was no significant difference in mortality among genotypes with respect to the abrasion treatment alone (Fig. 1A); we only found evidence for differential mortality among genotypes to the biotic challenges (Fig. 1B). We performed stepwise Akaike information criterion analysis of Cox proportional hazards models to determine which experimental factors and/or interactions affected mortality (Supplementary Table S1). Coral genotypes differed significantly in their mortality rates under bacterial challenge (Fig. 1B; p < 0.001). Bacterial treatment significantly increased mortality (p < 0.001) regardless of the genotype (*i.e*., mortality under bacterial challenge was modeled as mortality under abrasion amplified by the same factor in all genotypes). The increased mortality was similar between challenge agents (*V. diazotrophicus*: 5.7-fold; *V. owensii*: 3.7-fold), and there was no significant difference in time-dependent mortality between the two bacterial species (Supplementary Fig. S2; p = 0.108). Although abrasion clearly led to increased mortality (Supplementary Fig. S2), it did not conform to the assumptions of the Cox proportional hazards model (p < 1e-6 for model conformity) since it affected mortality only in the first several days, violating the assumption of risk invariance throughout the experiment. To nevertheless account for variation in mortality associated with abrasion, we ran our Cox proportional hazards model with abrasion included as a data stratification variable.

Based on the lack of difference between two *Vibrio* species we simplified our subsequent analyses by considering only two bacteria-related factor levels where corals were either exposed to bacteria (n = 12 per genotype, pooling *V. owensii* and *V. diazotrophicus* treatments) or not (n = 6 per genotype). Also, since bacterial challenge led to statistically indistinguishable multiplicative increases in mortality among genotypes (about 4–6 fold), we focused our study on variation in overall genotype robustness, expressed as the fraction of replicate fragments surviving until the end of experiment. As two extremes, >90% of all fragments of genotype 9 died in response to the experiment while genotype 30 experienced no mortality at all. The remaining six genotypes experienced intermediate mortality (42–75%). This distribution, with few individuals showing extreme values while the majority shows intermediate values, is expected of a natural quantitative trait following a normal distribution.

### Higher constitutive immune activities and responses do not translate into lower mortality rates

Phenoloxidase (PO) and prophenoloxidase (PPO) measurements serve as proxies for cytotoxic defenses via the melanin-synthesis pathway, and catalase (CAT) and peroxidase (POX) activities indicate antioxidant capabilities. CAT, POX and PO were expected to increase as a result of infection, while PPO was expected to decrease due to conversion to PO. We measured these activities in asymptomatic bacteria-challenged and unchallenged fragments sampled at the conclusion of the experiment. The data were analyzed using Markov Chain Monte Carlo-based Bayesian linear mixed model with two fixed factors: abrasion (yes or no) and bacterial challenge (challenged or control). Genotype-specific means and genotype-specific changes in activity in response to bacteria (reaction norms) were modeled as random effects. The estimates of genotype-specific effects were sampled 1000 times from the posterior distribution of parameters and tested for the sign of correlation with survival. The proportion of sampled parameter series exhibiting a particular sign of correlation can be interpreted as a posterior probability of correlation with that sign.

**Figure 2:**
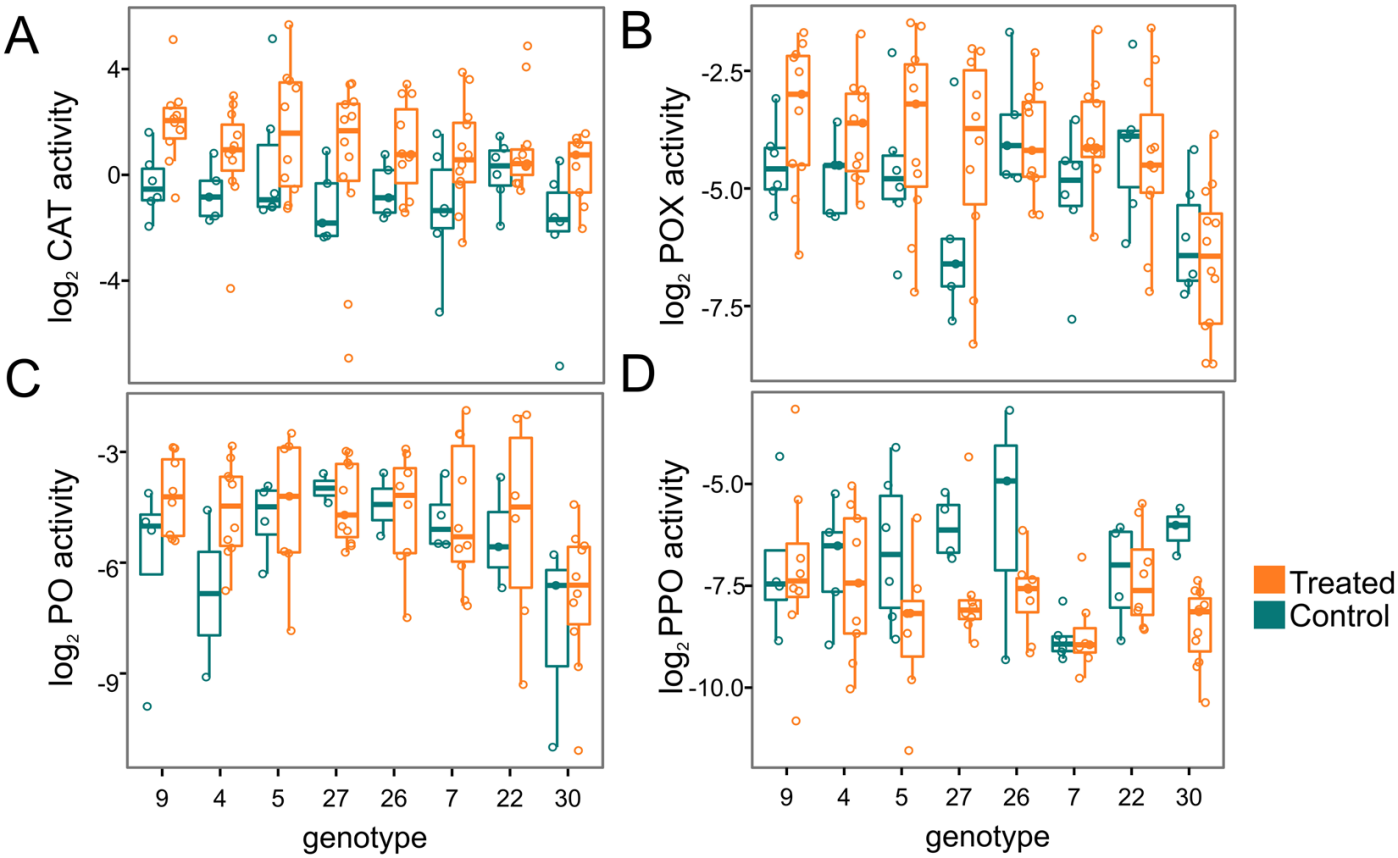
Immunity-related enzyme activities by genotype. Catalase (CAT), peroxidase (POX), phenoloxidase (PO), and prophenoloxidase (PPO) activities are represented as log_2_-transformed Δ absorbance mg protein^−1^ min^−1^. Genotypes are sorted by increasing survival from left to right.

**Figure 3:**
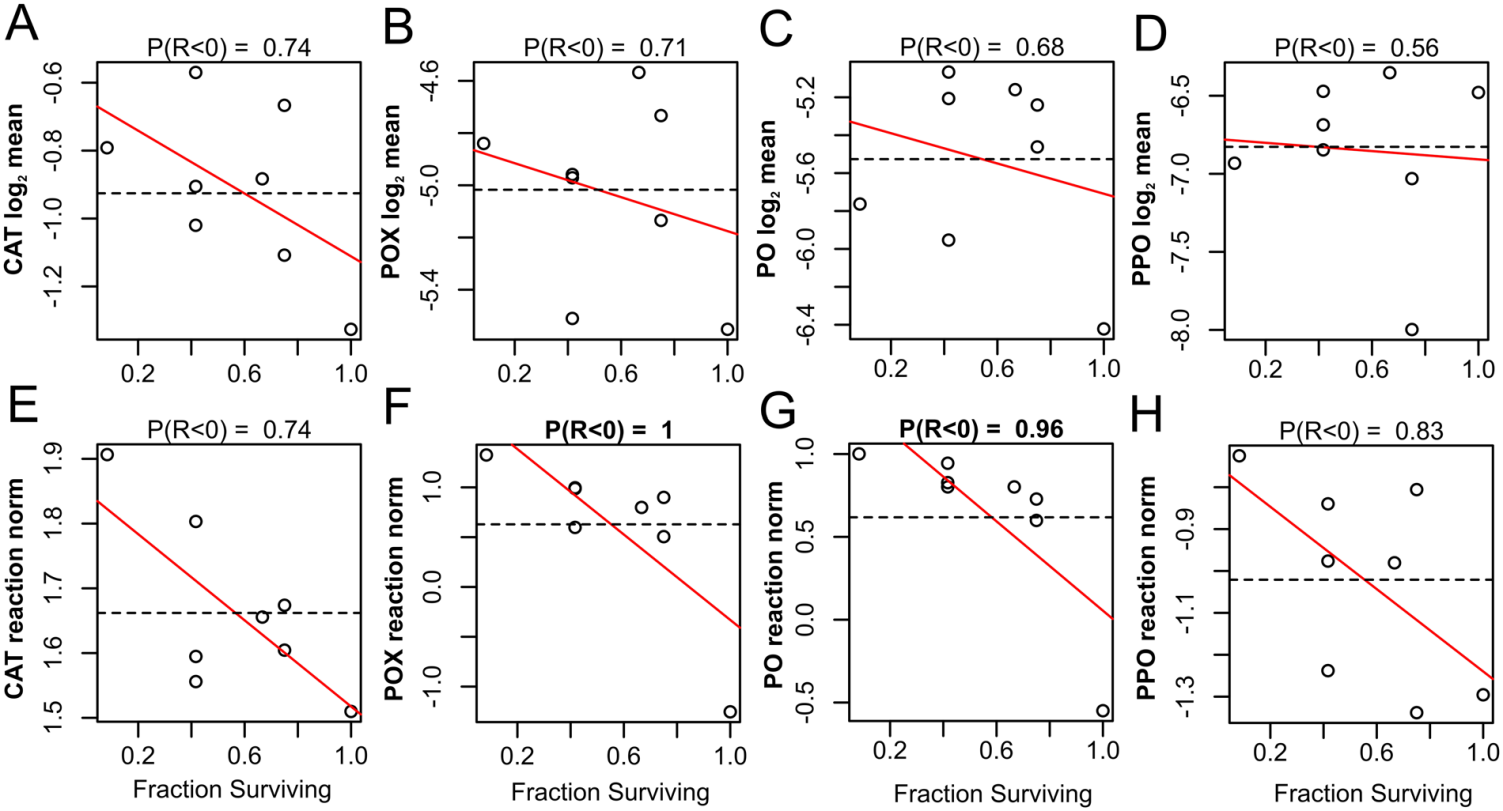
Correlations of mean immunity-related enzyme activities (**A–D**) and their reaction norms in response to bacterial challenge (**E–H**) with survival across genotypes. Mean catalase (CAT), peroxidase (POX), phenoloxidase (PO), and prophenoloxidase (PPO) activities are represented as log_2_-transformed Δ absorbance mg protein^−1^ min^−1^. Each point represents a posterior mean of the parameter for a genotype; dotted lines represent means across genotypes, red line is the linear model fit with survival as predictor variable. Value above the graph indicates posterior probability that the correlation with survival is negative.

All observed changes in immune activity were in the expected direction. Although pronounced changes in immunity-related enzyme activities were apparent as a result of bacterial challenge in some genotypes (Fig. 2), only the 3.2-fold increase in CAT activity was significant overall (Fig. 2A; P_MCMC_ = 0.002). POX and PO activities both increased ~1.5-fold under bacterial challenge (Fig. 2B,C), while PPO decreased 2-fold (P_MCMC_ = 0.06, Fig. 2D). Notably, for all enzymes, low-mortality genotypes tended to have lower means and reaction norms (Fig. 3). This negative correlation was significant (posterior probability > 0.95) for POX and PO reaction norms (Fig. 3F,G), implying weaker responses of these enzymes to bacterial treatment in more robust genotypes. These correlations were largely driven by the most robust genotype (30, no mortality under any treatment). Significant genotype correlations disappeared after excluding this one genotype (Supplementary Fig. S3).

### Sequencing results

Sequencing data have been deposited to the National Center for Biotechnology Information’s Short Reads Archive under accession numbers SRP074065 and SRP073937. Genome-wide gene expression was analyzed using TagSeq, a cost-efficient highly accurate alternative of RNA-seq^27, 28^. Sequencing yielded an average of 2,042,678 reads per sample. Because only one readable fragment per transcript is generated in TagSeq, each read represents an observation of a unique transcript (a “unique transcript count”, UTC) after removing PCR duplicates. A total of 44,687 genes were detected after mapping to the *A. millepora* transcriptome^29^. UTCs for all isoforms of a given gene were summed. An average of 360,642 UTCs per sample was obtained. The expression dataset was restricted to genes with a mean UTC greater than three, retaining 14,633 genes. No outlier samples were detected.

### Bacterial treatment triggered gene expression response in high-mortality corals

Only control and asymptomatic bacteria-challenged fragments sampled at the conclusion of the experiment were included in the gene expression analysis. Gene expression was analyzed using DESeq2 models including bacterial treatment (treated or control), percent survival (continuous variable), the interaction between the survival and bacterial treatment (*i.e*., survival-dependent effect of bacteria) and abrasion (yes or no) as a covariate. In a separate set of models, we also performed pairwise comparisons among all three bacterial treatments (control, *V. diazotrophicus*, and *V. owensii*). Only one gene was differentially expressed between the *V. diazotrophicus* and *V. owensii* treatments, further justifying pooling of these treatments as a general bacterial challenge.

**Figure 4:**
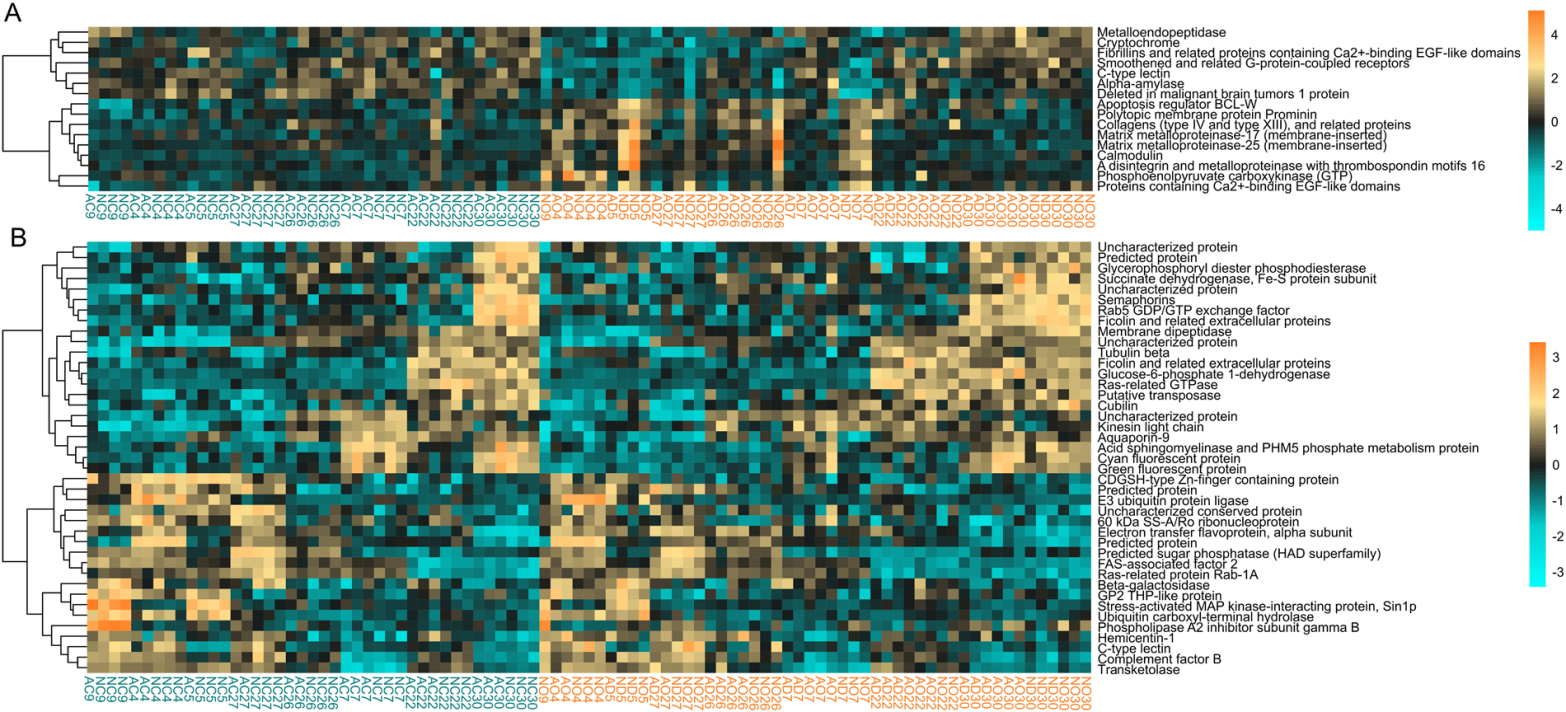
Gene expression differences in response to bacterial challenge (**A**, FDR = 0.01) and associated with survival rate among genotypes (**B**, FDR = 1e-4). In all panels, rows are genes and columns are samples. Samples are ordered by genotype survival, from lowest to highest, within each treatment group. The color scale is in log_2_-fold change relative to the gene’s mean. Genes are hierarchically clustered based on Pearson’s correlations of expression across samples. Bacteria-challenged samples are indicated in orange font below the heatmap; control samples are turquoise. Sample names correspond indicate abrasion (yes, “A”, or no, “N”), bacterial treatment (control, “C”, *V. diazotrophicus*, “D”, *V. owensii*, “O”), and genotype (number).

The contrast between control *vs*. bacteria challenge yielded 288 DEGs at FDR = 0.1. Testing for associations between gene expression and survival yielded 1412 DEGs at FDR = 0.1. To improve readability, we have only depicted the top 16 annotated DEGs for the effect of bacterial treatment (Fig. 4A; FDR = 0.01) and the top 41 annotated DEGs for the effect of survival (Fig. 4B; FDR = 1e-4). All gene expression results are included in Supplementary Data S1. Bacterial challenge triggered up-regulation of phosphoenolpyruvate carboxykinase (PEPCK) and several matrix metalloproteinases (MMPs) (FDR < 0.001; Fig. 4A). Interferon gamma (FDR = 0.07) and apoptosis regulator Bcl-W (FDR < 0.001) were upregulated in bacteria-challenged corals, whereas deleted in malignant brain tumors protein 1 (*dmbt1*; FDR = 0.002), cryptochrome (FDR = 0.004), carbonic anhydrase (only one out of 21 paralogous genes in this *A. millepora* transcriptome; FDR = 0.07), and galaxin (FDR = 0.04) were downregulated (Supplementary Data S1). Notably, the gene expression response to bacterial challenge was driven by higher-mortality genotypes, as expression profiles of the more robust corals (*i.e*., lower mortality) remained similar to the control condition (Fig. 4A). Indeed, a DESeq2 model predicted lower fold-changes in more robust corals for essentially all the bacteria-responding DEGs, implying minimal if any effect of bacterial challenge on more robust genotypes (Supplementary Fig. S4). Relative to less robust corals, challenged and unchallenged resistant corals expressed elevated glucose-6-phosphate 1-dehydrogenase and fluorescent proteins (cyan and green) and diminished stress-related MAP kinase-interacting protein, ubiquitin ligase, hemicentin-1, complement factor B, and C-type lectin (FDR < 0.001; Fig. 4B).

Tests of the interaction between survival and treatment yielded two DEGs at FDR = 0.1 (DESeq2 models have low statistical power detecting interaction terms involving a continuous variable). A rank-based gene ontology analysis revealed significant enrichment of terms including G-protein coupled receptor signaling pathway and protein ubiquitination, which were more upregulated in response to treatment in high mortality corals than in lower mortality corals. Ribosome biogenesis, small molecule metabolic process, protein folding, stress responses, and establishment of protein localization processes were less upregulated in response to treatment in high mortality corals compared to corals with lower mortality (Supplementary Fig. S5). No biological processes or molecular functions were significantly enriched with respect to the effect of treatment or survival.

### *Symbiodinium* profiles differ only by reef

*Symbiodinium* communities were profiled using RNA-seq reads mapping uniquely to clade A, B, C, or D *Symbiodinium* transcriptomes. On average we obtained 25,480 reads mapping to these transcriptomes per sample (maximum = 156,098, minimum = 4644). As expected for *A. millepora* in the northern region of the GBR, clade C dominated most genotypes^30^ but two colonies from Wilkie were dominated by D (Supplementary Fig. S6). There was no association of *Symbiodinium* clade dominance with survival rate (p = 0.33).

### Microbial community profiles differ between individuals and in response to bacterial challenge

The presence of *V. owensii* and *V. diazotrophicus* sequences was verified by NCBI BLAST queries (E-value cutoff 1e-100) using reference sequences for these strains (GenBank accession numbers GU018180^31^ and KF691569^32^, respectively). Overall, more *V. owensii* sequences were found (59 in untreated, 1920 in VO-treated, and 1115 in VD-treated). Only one diazotroph read was found in a control sample, whereas 350 and 45 were found in VD- and VO-treated samples, respectively. Clustering at 97% similarity identified 1238 operational taxonomic units (OTUs) based on the 16 S rRNA V4/V5 region. Two best-surviving genotypes (22 and 30) had significantly more cyanobacterial and chloroplast-derived OTUs than other genotypes (Supplementary Fig. S7; FDR = 0.001). Chloroplast OTUs were homologous to the green marine algae, Ulvophyceae, a group that includes common endolithic photoautotrophs^33^. The skeletons of genotypes 22 and 30 were noticeably green (Supplementary Fig. S7), supporting the hypothesis that elevated proportion of chloroplast OTUs in these corals is due to higher loads of endolithic algae. All chloroplast-derived OTUs were excluded from subsequent analysis of the bacterial populations. One sample that did have a lesion (NO53) was accidentally included in the sequencing, but removed from subsequent analysis. The microbiome of this one sample is substantially different from all other samples, yet *Vibrio* spp. are still noticeably absent. All OTU counts, including those from the sample with the lesion, are presented in Supplementary Data S2.

Faith’s phylogenetic diversity (PD_whole_tree) for each sample was calculated using the alpha_diversity.py script in QIIME. Generalized linear models tested the fixed effects of continuous survival (fraction) and treatment with reef, genotype, and number of sequences used to calculate the distances as random effects. Phylogenetic diversity of the microbial communities was greater in treated corals (effect = 2.35, p < 0.001) and in corals with lower mortality (effect = 2.51, p = 0.04) (Supplementary Fig. S8).

**Figure 5:**
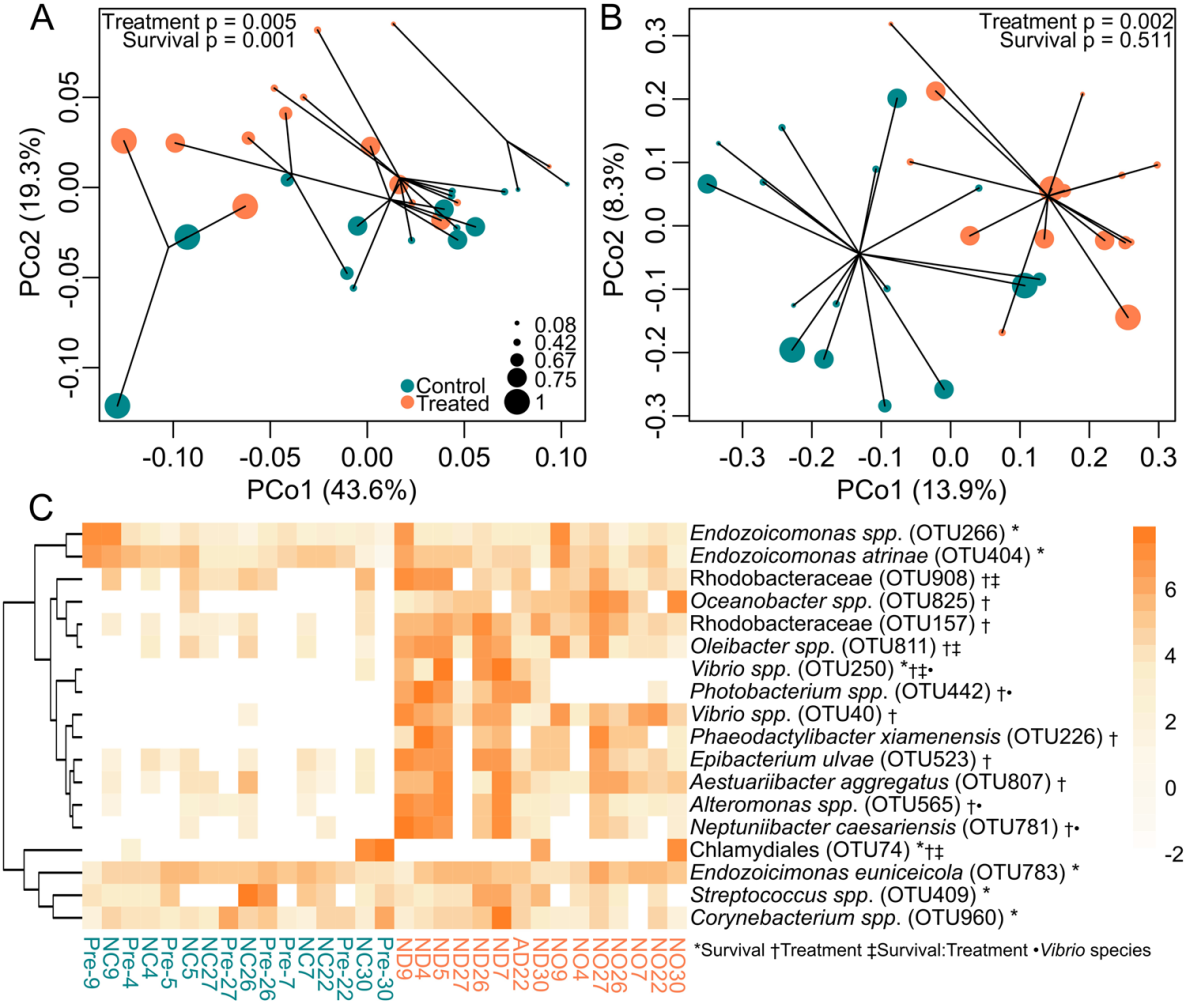
Microbial community composition by treatment and survival. Principal coordinate analysis of microbial community profiles using weighted (**A**) and unweighted (**B**) UniFrac distances. Samples are clustered according to the variable that separates along the major axis: survival fraction in (**A**) and by treatment in (**B**). P-values were generated by a PERMANOVA testing the effect of survival fraction and treatment. Sphere size represents survival fraction. (**C**) Heatmap of the most significantly (P_MCMC_ < 0.1) differentially abundant OTUs by treatment, survival, or their interaction. Symbols following OTU names indicate significance (see inset key). Color scale is in log_2_-fold change relative to the OTU’s mean. OTUs are hierarchically clustered based on Pearson’s correlations of abundance across samples. Samples are ordered by genotype survival, from lowest to highest, within each treatment group. Bacteria-challenged samples are indicated in orange font below the heatmap; control samples are turquoise. Sample names correspond indicate abrasion (yes, “A”, or no, “N”), bacterial treatment (control, “C”, *V. diazotrophicus*, “D”, *V. owensii*, “O”), and genotype (number). Samples labeled “pre-” were sampled before the experiment began.

Principal coordinate analysis (PCoA) of weighted (quantitative) UniFrac distances separated samples by survival along PCo1 and by treatment along PCo2 (Fig. 5A). Principal coordinates analysis based on unweighted UniFrac (qualitative) distances clustered samples only by treatment along the major axis (Fig. 5B). To investigate the relationship between host gene expression and microbial composition, we subset the RNAseq dataset for individuals with 16S sequencing data (n = 31) and modeled gene expression correlated with the first principal coordinate axis based on weighted UniFrac scores (Fig. 5A) after accounting for the effects of treatment and survival. A total of 123 genes were significantly differentially expressed with respect to the PCo1 axis (FDR = 0.1; Supplementary Data S3). GO enrichment analysis (Supplementary Fig. S9) suggested that elevated DNA repair and/or replication and macromolecule biosynthesis is associated with higher microbiome PCo1 values (samples that tended to have higher mortality) and elevated environmental sensing (GPCR signaling) is associated with lower PCo1 values (samples that tended to have lower mortality).

We used Poisson-lognormal generalized linear mixed models to test for significant differences in abundances in OTUs depending on treatment (bacteria-challenged *vs*. control), *Vibrio* species (control, *V. diazotrophicus*, and *V. owensii*), and survival (continuous variable), as well as the interaction between treatment and survival (Fig. 5C). Seven OTUs varied by survival fraction: *Vibrio spp*. (OTU250), *Streptococcus spp*. (OTU409), Chlamydiales (OTU74), *Endozoicomonas euniceicola* (OTU783), and *Corynebacterium spp*. (OTU960) were significantly more abundant in corals with higher survival over all treatments. Another two *Endozoicomonas* species (OTU266 and OTU404) were significantly positively associated with higher mortality. Thirteen OTUs were significantly differentially abundant by bacterial treatment: only one of these (Chlamydiales OTU74, which was only present in genotype 30) was less abundant in treated corals. Four of the OTUs that were significantly enriched by bacterial challenge differed by *Vibrio* species used: *Vibrio spp*. (OTU250), *Photobacterium spp*. (OTU442), *Alteromonas spp*. (OTU565), and *Neptuniibacter caesariensis* (OTU781) were all significantly more abundant in *V. diazotrophicus*-treated corals than in *V. owensii*-treated corals. Tests for the interaction of bacterial treatment and survival revealed that *Vibrio spp*. (OTU250), *Oleibacter spp*. (OTU811), and Rhodobacteraceae (OTU908) were all less abundant in treated corals with high survivorship than in corals with low survivorship.

### Diagnostic gene expression biomarker identification and validation

**Figure 6:**
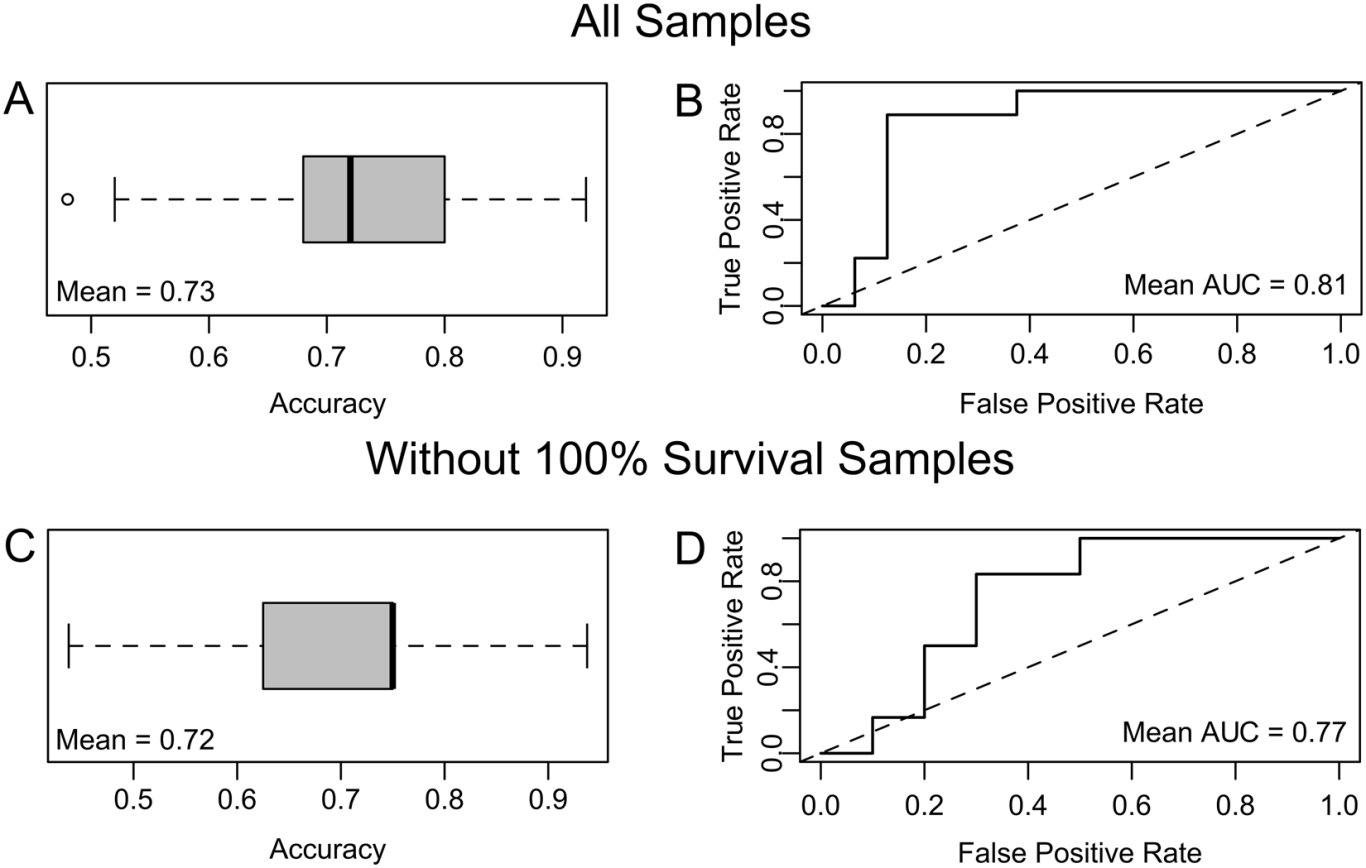
Double-gene biomarker predictive power. Average accuracy using a predicted value cutoff of 0.5 (**A**,**C**) and receiver operator characteristic (ROC) curves (**B**,**D**) for all data (top) and only corals with less than 100% survival (bottom) over 500 iterations. The mean area under the curve (AUC) indicates the sensitivity and specificity of the model. Common AUC score metrics are 0.90–1 = excellent, 0.80–0.90  =  good, 0.70–0.80 = fair, 0.60–0.70 = poor, and 0.50–0.60  = fail.

Two candidate survival-specific genes were selected based on their high and dynamic expression: deleted in malignant brain tumors protein 1 (*dmbt1*) and matrix metalloproteinase (*mmp*). *Dmbt1* expression was positively correlated with survival and *mmp* expression was negatively associated with survival in our gene expression analysis. Thus, a *Dmbt1*:*mmp* expression ratio greater than 1 indicates lower risk. This gene pair was used in a self-normalizing double-gene qPCR assay quantifying the log-ratio of expression of these two genes, *sensu* Kenkel *et al*.^34^. We quantified expression of these two genes in 74 samples from 19 genotypes from the independent validation experiment using qPCR. We measured the predictive power of the double-gene assay using logistic regression models testing binary survival outcomes: “low risk” corals had survival fractions exceeding 0.5 and all other corals were classified as “high risk.” The original (n = 50) and validation (n = 74) *Dmbt1*:*mmp* expression ratios and survival rates (n = 83 low risk; n = 41 high risk) were pooled and randomly split into training (80%) and testing (20%) subsets over 500 iterations. Risk values were predicted from the test dataset based on the logistic model fit to the training dataset using the *predict* function in R. Total accuracy was defined as the percent correct risk predictions using a cutoff of 0.5 on the predicted value. The area under the curve (AUC) of the receiver operating characteristic (ROC) curve was calculated using the R package *ROCR* ^35^. An AUC equal to 1 indicates a perfectly discriminatory test, whereas an AUC equal to 0.5 indicates that the test operates no better than chance alone. The average overall accuracy of the model was 73% (Fig. 6A). The average AUC was 81%, indicating good discriminatory power (Fig. 6B). Thus, using just the expression level of these two genes, reef scientists could distinguish asymptomatic corals that were likely to become sick from identical healthy corals with 73% accuracy using this model. Excluding any samples with 100% survival from the model only reduces the model accuracy to 72% and the AUC to 0.77 (Fig. 6C,D).

## Discussion

Differences in disease susceptibility across coral taxa have been attributed to baseline differences in constitutive immunity, where more resistant individuals invest in higher immune activity^25, 26^. Another study found that intraspecific differences in *Symbiodinium* composition correlated with coral disease susceptibility^36^. Here, we examined multiple aspects of holobiont physiology to investigate the physiological basis of colony-specific responses to a biotic challenge.

We found that individuals which experienced higher mortality also possessed higher antioxidant or cytotoxic activities, either as a baseline measure in the unchallenged fragments or in response to bacterial challenge (Fig. 2). In contrast, low-mortality corals tended to respond less to the bacterial challenge (Fig. 3), though this relationship was largely driven by a single genotype with 100% survival (Supplementary Fig. S3). The lack of positive correlation between immunity responses and survival suggests that mechanisms other than host immune activity contribute towards the observed variation in robustness to the bacterial challenge in our experiment.

Surprisingly few genes involved in stress responses or immunity were upregulated in low-mortality corals in response to bacterial challenge. Instead, these individuals exhibited a more “healthy” gene expression profile: they had elevated glucose-6-phosphate1-dehydrogenase expression that is important for cell growth^37^, as well as increased levels of fluorescent proteins whose abundances have been linked to health status in corals^38, 39^. Another signature of lower mortality was the diminished abundance of ubiquitination-related transcripts, *e.g*., ubiquitin ligases and ubiquitin carboxy-terminal hydrolases. Ubiquitination labels damaged proteins for removal and is a general hallmark of cellular stress^40^. Ubiquitin has been shown to be upregulated in heat-stressed corals with high levels of damaged proteins^41–43^. Differential expression of ubiquitination-related transcripts suggests that some genotypes may have been experiencing more baseline cellular stress than others throughout the experiment even though all genotypes were maintained in benign common conditions for over one month prior to and during the experimental procedure.

High-mortality corals exhibited abundant changes in gene expression in response to bacterial challenge, including upregulation of matrix metalloproteinases, which are also upregulated in naturally occurring coral disease and bleaching^38, 43^, and an apoptosis regulator (Fig. 4A). At the same time, gene expression in low-mortality corals responded to bacteria much less if at all (Fig. 4A and Supplementary Fig. S4). Taken together with the lack of elevated immune activity in low-mortality corals (Figs 2–3), our conclusion is that these corals survived better because they were generally less sensitive to the adverse effects of bacterial challenge, not because they launched a more robust response. To explore why these coral hosts were less sensitive to the bacterial challenge, we investigated members of the coral holobiont. Previous research suggests that corals hosting clade D *Symbiodinium* can be more resistant to disease than those hosting clade A symbionts^36^. The most robust genotype (30) was dominated by clade D, but so was a much weaker genotype (Supplementary Fig. S6). As only these two coral genotypes contained substantial proportions of clade D symbionts, our experiment cannot make firm conclusions concerning the effect of *Symbiodinium* clade on ability to withstand biotic challenge, but this area of investigation deserves further attention.

Although the proportion of *Vibrio* spp. was higher in challenged corals than in controls (Fig. 5C), these OTUs remained relatively rare, suggesting that the introduced *Vibrio spp*. were not the sole cause of lesion formation. This study is not the first instance wherein a simple “one pathogen = one disease” model fails to describe a coral lesion: microbiota of diseased corals often differ dramatically from the microbiota of healthy corals, suggesting that more than one bacterium is involved in deteriorating health^10^. Likewise, healthy corals often harbor “pathogenic” microbes^8^. The “pathobiome” concept has been recently introduced to the field of coral disease biology to explain these apparent contradictions^12^. The pathobiome describes interactions between pathogenic microbes and healthy microbiota that contribute towards disease processes^13^. Here, *Vibrio* treatment caused an increase in the abundance of taxa previously reported to be associated with disease and stress in marine organisms, including Alteromonadaceae^11, 44^, Pseudoalteromonadaceae^11^, Rhodobacteraceae^45, 46^, and, expectably, Vibrionaceae^11, 47–49^, suggesting that the introduced bacteria may have triggered a disturbance in the coral-associated microbiome that facilitated the proliferation of multiple bacterial species which contributed to the disease outcome (*i.e*., the pathobiome). To characterize the physiological consequences of harboring these distinct microbial communities in the host, we modeled gene expression by PCo1 value of the weighted UniFrac analysis (Fig. 5A). This analysis revealed differences in host gene expression that are associated with differences in microbial community membership, while controlling for the effects of survival and treatment. Major differences included stress responses, nucleic acid processing, and cell–cell signaling (Supplementary Fig. S9). These differentially expressed genes provide insight into the physiological consequences of harboring these distinct microbiomes and/or serve as targets for future investigations of genes that may modulate host–microbiome interactions.

The most striking difference in microbial composition is that the two best-surviving corals (genotypes 22 and 30) harbored high proportion of chloroplast-derived OTUs (Supplementary Fig. S7). These OTUs are homologous to various cyanobacteria and Ulvophyceae, an abundant bioeroding marine algae^50^, suggesting the presence of endolithic algae or cyanobacteria that would also explain why the skeletons of these genotypes were noticeably green (Supplementary Fig. S7). It is tempting to speculate that these microbes facilitate host defense by actively secreting antimicrobial compounds, an ability that has been well characterized in cyanobacteria^51, 52^. This putative association between chloroplast-derived OTU abundance and lower mortality under bacterial challenge merits further investigation.

This study developed and validated a double-gene assay^34^ predicting whether an asymptomatic coral will develop disease lesions in the near future (Fig. 6), which can be used to assess mortality risks during coral disease outbreaks. The putative roles of the genes forming the assay provide important insights into potential mechanisms underlying coral disease susceptibility. *Deleted in malignant brain tumors 1* (*dmbt1*) is found in the gut mucosa of humans where it acts as a pattern recognition receptor that maintains mucosal homeostasis by inhibiting bacterial invasion and suppressing inflammation^53, 54^. Other transcriptomic studies have found that *dmbt1* was downregulated in oysters upon bacterial challenge^55^, upregulated in the *Symbiodinium*-hosting coral *Orbicella faveolata* after lipopolysaccharide challenge^56^, and upregulated in aposymbiotic sponges compared to sponges infected with clade G *Symbiodinium*, suggesting that *dmbt1* may play a role in mediating various marine symbioses^57^. Elevated *dmbt1* in all control fragments and in the low-mortality corals relative to high-mortality, bacterial-challenged corals may signify the role of this protein in maintaining healthy stable symbiotic associations with commensal microbes. The diagnostic gene that was regulated in the opposite direction, a *matrix metalloproteinase* (*mmp*), belongs to a family of enzymes with a wide range of functions. The upregulation of MMPs in response to parasitic protists in a gorgonian coral^58^ and in *A. hyacinthus* affected with White Syndrome-like symptoms^38^ suggests an active role of these proteins in the immune response of cnidarians. Changes in *dmbt1* and *mmp* may represent some of the earliest coral responses to immune challenge, as they are detectable even in asymptomatic corals.

We found that neither introduced *Vibrio* species proliferated within the coral host in sampled asymptomatic fragments, but both bacterial treatments triggered the rise of putative opportunistic pathogens in the coral microbiome and subsequent development of disease lesions in corals that exhibited less healthy gene expression profiles. We are not be the first to argue that a coral disease can be caused by opportunistic infection exploiting a compromised host^7^, and many coral diseases are associated with broad shifts in microbial community composition beyond the rise of a single pathogen^10, 11, 59^. If coral diseases in nature similarly arise because of weaknesses in holobiont physiology, instead of the virulence of any single etiological agent, environmental stressors compromising coral condition might play a larger role in disease outbreaks than is currently thought.

## Methods

### Coral collection

Corals in the experiment were collected under the permit number G12/35236.1 by the Great Barrier Reef Marine Park Authority of Australia. *A. millepora* were sampled from 3–6 m depth at the lagoon at Lizard Island (14°41′13.64 S:145°27.75E) and the sheltered side of Wilkie Island (13°46′43.33 S:143°38.75E; n = 4 colonies per reef) in the Great Barrier Reef in October 2013. Each colony is hereafter referred to as an individual genotype, a classification that is confirmed by our transcriptomic results that clearly distinguish separate colonies (genotypes). Colonies were maintained in an outdoor raceway under flow through conditions and filtered natural light at the National Sea Simulator at the Australian Institute of Marine Science until fragmentation in November 2013. Colonies were further fragmented (Fig. S1) into replicate nubbins (4–5 cm) and secured on wire hooks (n = 18 per genotype).

### Experimental aquaria and abrasion procedure

Coral fragments were secured upright in individual jars containing 200 mL 0.04 μM-filtered seawater (FSW). Fluorescent lights provided light on a 12:12 h day/night schedule and the temperature was maintained at 26–27 °C. Half of the 18 coral fragments received two small (~1 cm^2^) abrasions with a high-pressure airgun. These small abrasions mimicked clean lesion-associated injuries that occur in nature (*e.g*., corallivorous fish bites). The original purpose of the abrasion was to increase the probability of lesion development after bacterial challenge, in the event that inoculation with bacteria alone did not affect the corals. In reality both abraded and non-abraded corals developed lesions, thus all treatments were included in the analysis to maximize statistical power.

### Bacterial culturing and challenge

*Vibrio owensii* strain DY05 and a diazotroph with high sequence similarity to *V. diazotrophicus* were used in this study. These bacteria were chosen based on their published pathologies (or lack thereof) and the availability of local isolates to minimize biocontainment risks. *V. owensii* has been implicated as the pathogenic agent of a tissue-loss disease in a Hawaiian coral, *Montipora capitata* ^5^. This isolate of *V. owensii* had been recently sampled during an infectious disease outbreak in cultured lobsters at the research facility. At the time of this study *V. diazotrophicus*, a nitrogen-fixing bacterium that had been isolated from healthy *A. millepora* juveniles^32^, had not been implicated as a causative agent of any coral disease. Single isolates of each bacterial strain were recovered from glycerol stocks on Difco Marine Agar-2216 (BD, Franklin Lakes, NJ, USA) at 28 °C. Cultures were incubated overnight at 28 °C with shaking (150 rpm) in Difco Marine Broth-2216 (BD). Overnight cultures were triple-washed in FSW by centrifugation at 5000 *g* for ten minutes and resuspended in FSW. Washed cells were diluted to a final concentration of 1 × 10^6^ colony forming units (CFUs) · mL^−1^ in FSW. Cell densities were determined by counting CFUs resulting from plated serial dilutions and constructing a cell density calibration curve of absorbance (595 nm) versus CFU number. Of the nine abraded fragments per genotype, three were challenged with *V. owensii*, three were challenged with *V. diazotrophicus*, and three received daily “inoculations” of FSW (control). The nine non-abraded fragments received the same treatments. Aquarium water was changed daily preceding each bacterial challenge. Fragments were monitored for tissue loss and photographed twice daily throughout the experiment with a Nikon D300 digital camera (Nikon, Tokyo, Japan). Corals were photographed and removed from the experiment when tissue loss was visually estimated at 50% or more of the total surface area of the fragment (*e.g*., Fig. S7A: Genotypes 9 and 26). Symptomatic fragments (*i.e*., those that developed a lesion) were not included in subsequent analyses. All remaining asymptomatic fragments were frozen in liquid nitrogen and stored at −80 °C on the sixth day following the initial bacterial challenge for subsequent gene expression and protein analyses.

### Survival analysis

All statistical analyses were performed in R version 3.3.1^60^. The time when fragments suffered ~50% tissue loss was recorded for each fragment as time of death. Survivorship analyses were performed for each genotype, reef, abrasion treatment, and bacterial challenge using the Kaplan-Meier estimate of the survival function as implemented by *survfit* in the R package *survival* ^61^. Step-AIC analysis based on Cox proportional hazards model was performed using *stepAIC* function of the *MASS* package and *coxph* function of the *survival* package. The full model tested involved abrasion (abrasion/no abrasion), bacteria (control, *V. owensii*, *V. diazotrophicus*), genotype (eight categories) and the interaction between bacteria and genotype. The selected model with the lowest AIC score retained all these factors except the bacteria:genotype interaction, which increases the AIC score by 3 points. Testing for conformity of these factors to the model assumptions using the *cox.zph* function of package *survival* revealed that abrasion was not suitable as a factor; it was therefore modeled as a data stratification variable using the *strata* function of the *survival* package.

### Enzymatic assays

Coral proteins were extracted following a protocol adapted from established procedures^62^. Briefly, coral tissue was removed using an airbrush and cold extraction buffer (100 mM Tris-HCl, pH 7.8, with 0.05 mM dithiothreitol). Airbrushed tissue slurries were homogenized with 1 mm glass beads (BioSpec, Bartlesville, OK, USA) by vortexing for two minutes. The tissue slurry was centrifuged at 4 °C for 10 minutes at 3200 *g* to separate coral and algal fractions. The coral protein supernatant (protein extraction) was removed and stored at −80 °C until use. Surface area determinations of airbrushed skeletons were made following a modified wax dipping protocol^63^. A standard curve was prepared from a series cylinders of known surface area dipped in paraffin wax (Gulf Wax, Roswell, GA, USA) at 60 °C. Coral skeletons were weighed and dipped twice in paraffin wax (59–60 °C), weighing after each wax dip. For each fragment, the difference between initial weight and weight after second dip was compared to the standard curve to yield surface area in cm^2^.

Total protein was assessed in triplicate using the RED660 protein assay (G Biosciences, St. Louis, MO, USA) with a standard curve prepared from bovine serum albumin. Sample absorbance at 660 nm was compared to the curve and normalized to surface area and the tissue slurry volume.

Prophenoloxidase activity was assayed in triplicate by mixing 20 μL of sodium phosphate buffer (50 mM, pH 7.0), 25 µL of trypsin (0.1 mg · mL^−1^), and 20 μL of protein extract. Dopamine (30 μL, 10 mM) was added as substrate and absorbance at 490 nm was measured every 30 seconds for 15 minutes. Change in absorbance was calculated during the linear range of the curve (1–3 minutes). Activity was expressed as the change in absorbance per mg of protein (∆A_490_ · mg protein^−1^ · min^−1^). Phenoloxidase activity was assayed in triplicate by mixing 20 μL of sodium phosphate buffer (50 mM, pH 7.0), 25 μL of sterile water, and 20 μL of protein extract. Dopamine (30 μL, 10 mM) was added as substrate and absorbance at 490 nm was measured every 30 seconds for 15 minutes. Change in absorbance was calculated during the linear range of the curve (1–3 minutes). Activity was expressed as the change in absorbance per mg of protein (∆A_490_ · mg protein^−1^ · min^−1^). Catalase activity was assayed in triplicate by mixing 45 μL of sodium phosphate buffer (50 mM, pH 7.0), 75 μL of 25 mM H_2_O_2_, and 5 μL of protein extract. Samples were loaded on ultraviolet transparent plates (UltraCruz, Santa Cruz Biotechnology, Dallas, TX, USA) and absorbance at 240 nm was measured every 30 seconds for 15 minutes. Change in absorbance was calculated during the linear range of the curve (1–3 minutes). Activity was expressed as the change in hydrogen peroxide concentration per mg of protein (∆H_2_O_2_ · mg protein^−1^ · min^−1^). Peroxidase activity was assayed in triplicate by mixing 40 μL of sodium phosphate buffer (10 mM, pH 6.0), 25 μL of 10 mM guaiacol, and 10 μL of protein extract. Absorbance at 470 nm was measured every 30 seconds for 15 minutes. Change in absorbance was calculated during the linear range of the curve (1–3 minutes). Activity was expressed as the change in absorbance per mg of protein (∆A_470_ · mg protein^−1^ · min^−1^).

Activities of CAT, POX, PO, and PPO were normalized to the total protein concentration, log-transformed, and compared among treatments using *MCMCglmm* function^64^. Log-transformation was chosen based on diagnostic plots of a linear model with abrasion, bacteria and genotype as factors. The MCMC model included abrasion and bacterial treatment as fixed factors and genotype-specific mean and reaction norm in response to bacteria as random effects. The reaction norm was calculated as the change in enzymatic activity for each genotype. We used the standard weakly informative inverse-Wishart priors for the random effects. The model was run for 55,000 iterations collecting parameter samples every 50 iterations; parameters collected during the first 5000 iterations were discarded.

### Gene expression

All tissue samples for gene expression were collected from asymptomatic individuals within one hour on the sixth day following the initial bacterial challenge. RNA was extracted from whole preserved coral nubbins (including any mucus, holobiont tissue containing intracellular algal symbionts, and skeleton) using RNAqueous Total RNA Isolation kits (Ambion). Genome-wide gene expression was analyzed using tag-based RNA-seq (TagSeq) method^27^. The reads were trimmed, deduplicated, quality filtered, mapped to the *A. millepora* reference transcriptome^29^ using *bowtie2* ^65^, and converted to UTCs representing the number of independent observations of a transcript of a specific gene, summed over all isoforms for each gene. Such read processing in TagSeq was recently shown to result in more accurate representation of transcript abundances than standard RNA-seq^28^. Sample outliers were detected using R package the *arrayQualityMetrics* ^66^ and differential gene expression analysis was performed using *DESeq2* ^67^. P-values for significance of contrasts between treatments, survival, and the survival by treatment interaction were generated based on Wald statistics and adjusted for multiple testing using the Benjamini-Hochberg method^68^ applied following an independent filtering procedure (integral to the DESeq2 pipeline) to maximize the number of detected differentially expressed genes. Gene expression heatmaps with hierarchical clustering of expression profiles were created with the *pheatmap* package in R^69^.

### Symbiodinium analysis

Trimmed and quality filtered RNAseq reads were mapped to *Symbiodinium* clade A, B, C, and D transcriptomes with *bowtie2* ^65^. A custom perl script (zooxType.pl, Supplemental File 1) generated counts from mapped reads and calculated clade fractions. The R package *MCMC.OTU* ^70^ was used to implement generalized linear mixed model analysis to test for significant differences in clade abundances.

### Microbial community analysis

Microbial communities were profiled for one pre-treatment (separated from the colony before the experiment began), *V. diazotrophicus*-treated, *V. owensii*-treated, and untreated control fragment for each genotype. DNA was isolated using an RNAqueous kit together with the RNA for gene expression analysis. DNA samples were diluted to 10 ng · µL^−1^. The bacterial 16 S rRNA gene V4/V5 region was amplified using the Hyb515F (5′-TCGTCGGCAGCGTCAGATGTGTATAAGAGACAGGTGYCAGCMGCCGCGGTA -3′) and Hyb806R (3′-TAATCTWTGGGVHCATCAGGGACAGAGAATATGTGTAGAGGCTCGGGTGCTCTG-5′) primers and sequenced on the MiSeq V2 platform to generate 250 bp paired reads. Sequences with homopolymer runs of six or more consecutive bases (12,128 sequences) or incorrect primer sequence (63,719 sequences) were discarded using split_libraries.py in QIIME (Quantitative Insights Into Microbial Ecology)^71^. Sequences of 97% similarity were clustered into operational taxonomic units (OTUs). A phylogeny was generated by aligning representative sequences that were filtered to remove gaps and hypervariable regions. Principal coordinate analyses and PERMANOVA (*adonis*) were conducted based on UniFrac distances^72^ using the R package *vegan* ^73^. Significant differences in abundances of OTU types between treatment and continuous survival were assessed using generalized linear mixed model implemented in the MCMC.OTU package in R^70^.

### Validation experiment

The bacterial challenge was repeated in March 2013. *A. millepora* (N = 43 genotypes, five fragments per genotype) were challenged daily with 10^6^ CFU · mL^−1^ *V. owensii* DY05 as described above. An equal number of control fragments for each genotype were maintained under ambient conditions (26 °C). Survival was monitored for seven days of daily bacterial challenges. On the final day, ~1 cm^2^ tissue samples were preserved in 100% ethanol and stored at −20 °C. These corals were collected under the permit number G12/35236.1 by the Great Barrier Reef Marine Park Authority of Australia. Nineteen genotypes spanning a range of survival rates were used in the qPCR validation.

### Quantitative real-time PCR (qPCR) validation of putative biomarkers

Candidate diagnostic gene expression biomarkers were selected based on differential expression with regard to survival in the response gene expression dataset and had putative functions that could be related to immune defense. Primers were designed using Primer3^74^. For *dmbt1*, the forward and reverse primers were 5′-TCATGTGACCTGTGTTGGGA-3′ and 5′-GGTGACGCTCCGATCAAAC-3′, respectively. For *mmp*, the forward and reverse primers were 5′-GTTCCAAAATCGGCCACACC-3′ and 5′-CGTTATGCAGGGCTTCCAGA-3′, respectively. Primer pair specificity was verified by gel electrophoresis and melt curve analysis of the amplification product obtained with template *A. millepora* cDNA. Primer efficiencies were determined by amplifying a series of two-fold dilutions of *A. millepora* cDNA and analyzing the results using function *PrimEff* of the *MCMC.qpcr* package in R^75^. Briefly, C_T_ (threshold cycle) results were plotted as C_T_ vs. log_2_[cDNA], and amplification efficiencies (amplification factor per cycle) of each primer pair were derived from the slope of the regression using formula: efficiency = 2^−(1/slope)^ ^76^. RNA isolation, cDNA preparation, and qPCR were carried out as previously described^34^ with the exception that the RNAqueous Total Isolation Kit (Ambion) was used to isolate total RNA. Linear regression implemented in R was used to test for the relationship between survival fraction and the log-difference in expression between the two candidate genes, as in ref. 34.

## Electronic supplementary material

Supplementary Figures and Legends

Supplementary Data 1

Supplementary Data 2

Supplementary Data 3

Supplemental File 1
