# Supplementary material for: Intraspecific differences in molecular stress responses and coral pathobiome contribute to mortality under bacterial challenge in Acropora millepora
Source: Sci Rep. 2017 Jun 1;7:2609. doi: 10.1038/s41598-017-02685-1 (PMC5454005; doi:10.1038/s41598-017-02685-1)
Supplement: Supplementary file 1 — Supplementary Figures and Legends [file 41598_2017_2685_MOESM1_ESM.docx]

**SUPPLEMENTARY INFORMATION**

**Intraspecific differences in molecular stress responses and coral pathobiome contribute to mortality under bacterial challenge in *Acropora millepora***

Short title: Signatures of mortality risk in corals

Rachel M. Wright^1,*^, Carly D. Kenkel^2^, Carly E. Dunn^1^, Erin N. Shilling^1^, Line K. Bay^2^, Mikhail V. Matz^1^

^1^Department of Integrative Biology, University of Texas at Austin, 205 W. 24^th^ Street C0990, Austin, TX 78712, USA.

^2^Australian Institute of Marine Science, PMB No. 3, Townsville MC, Queensland 4810, Australia.

*Corresponding author:

Rachel M. Wright

University of Texas at Austin

205 W. 24^th^ Street C0990

Austin, TX 78712, USA

Phone: (512) 475-6426

E-mail: rachelwright8@gmail.com

**
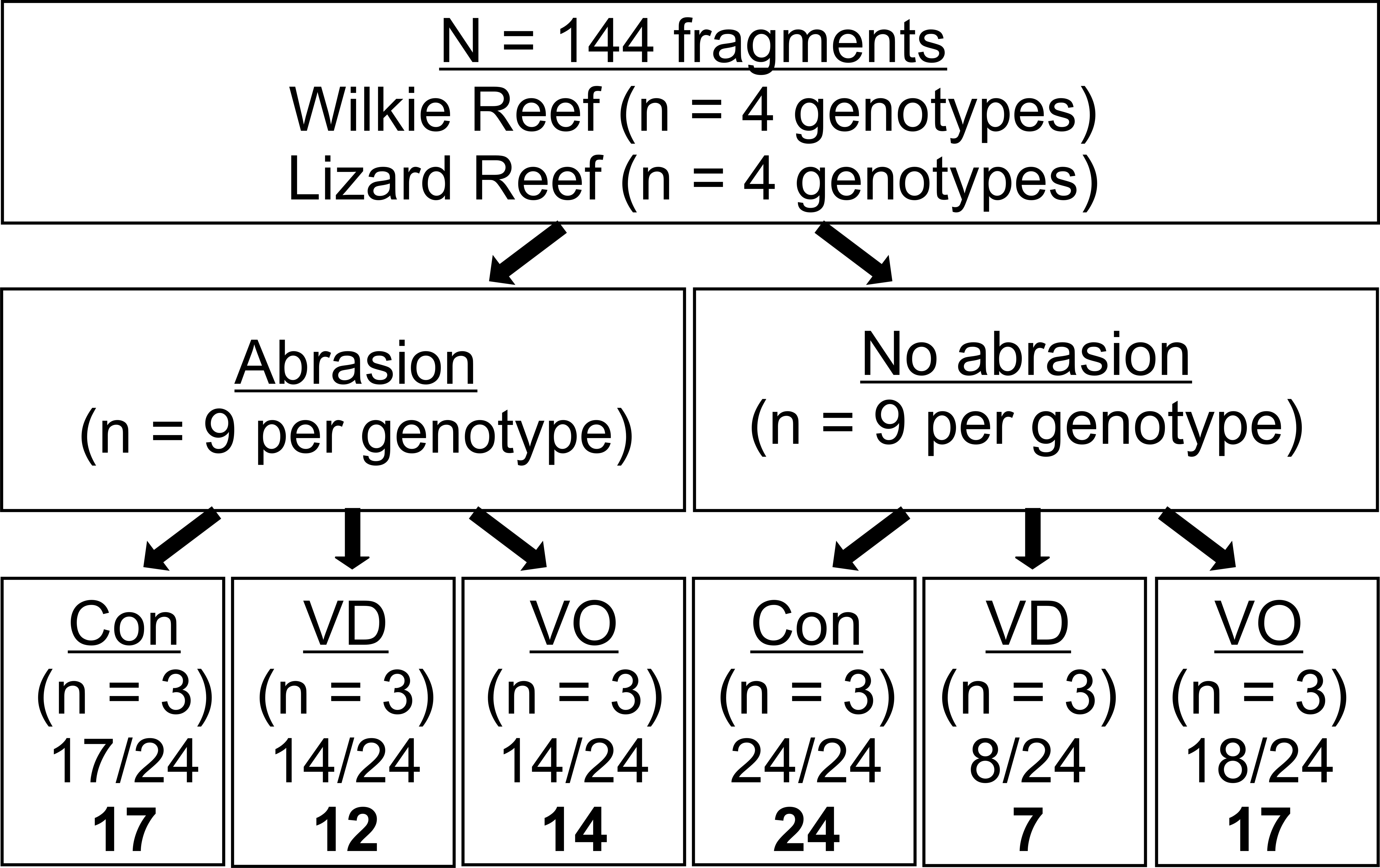
**

**Fig. S1: Experimental design.** Eight genotypes (four from Lizard Island, four from Wilkie Island) were separated into 18 fragments, half of which were abraded. Three fragments from each abrasion treatment received filtered seawater as a control (Con), *Vibrio diazotrophicus* (VD), or *V. owensii* (VO). Fractions indicate the number of corals alive at the end of the experiment in each treatment (n = 24 per treatment). Bolded numbers indicate the number of samples included in the gene expression analysis after expression outliers were removed.


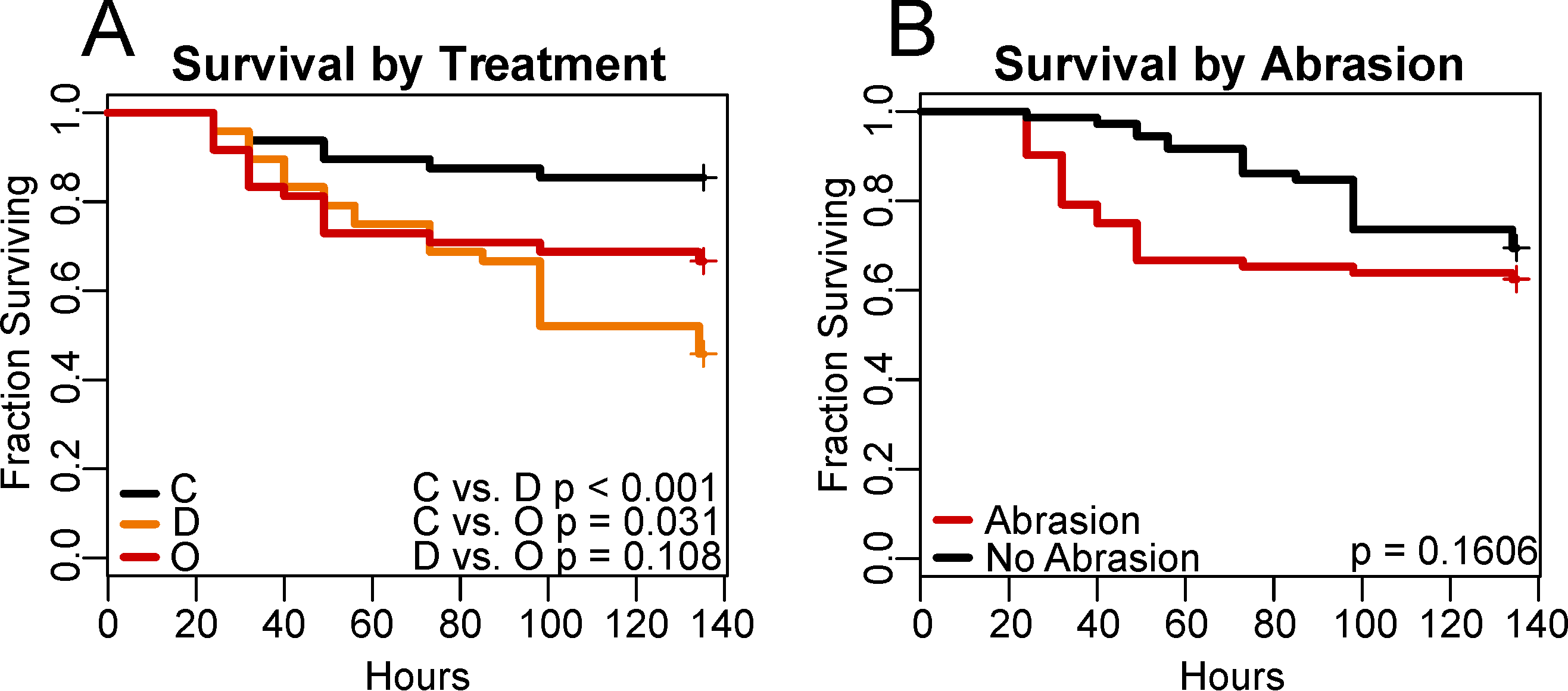


**Fig. S2: *A. millepora* survival by treatment during bacterial challenge.** (**A)** Black, orange, and red lines represent the survival of control, *V. diazotrophicus*-challenged, and *V. owensii*-challenged corals, respectively. **(B)** Black and red lines represent the survival of corals that abraded and non-abraded corals, respectively. P-values were generated by Cox proportional hazards models testing the effect of each treatment.


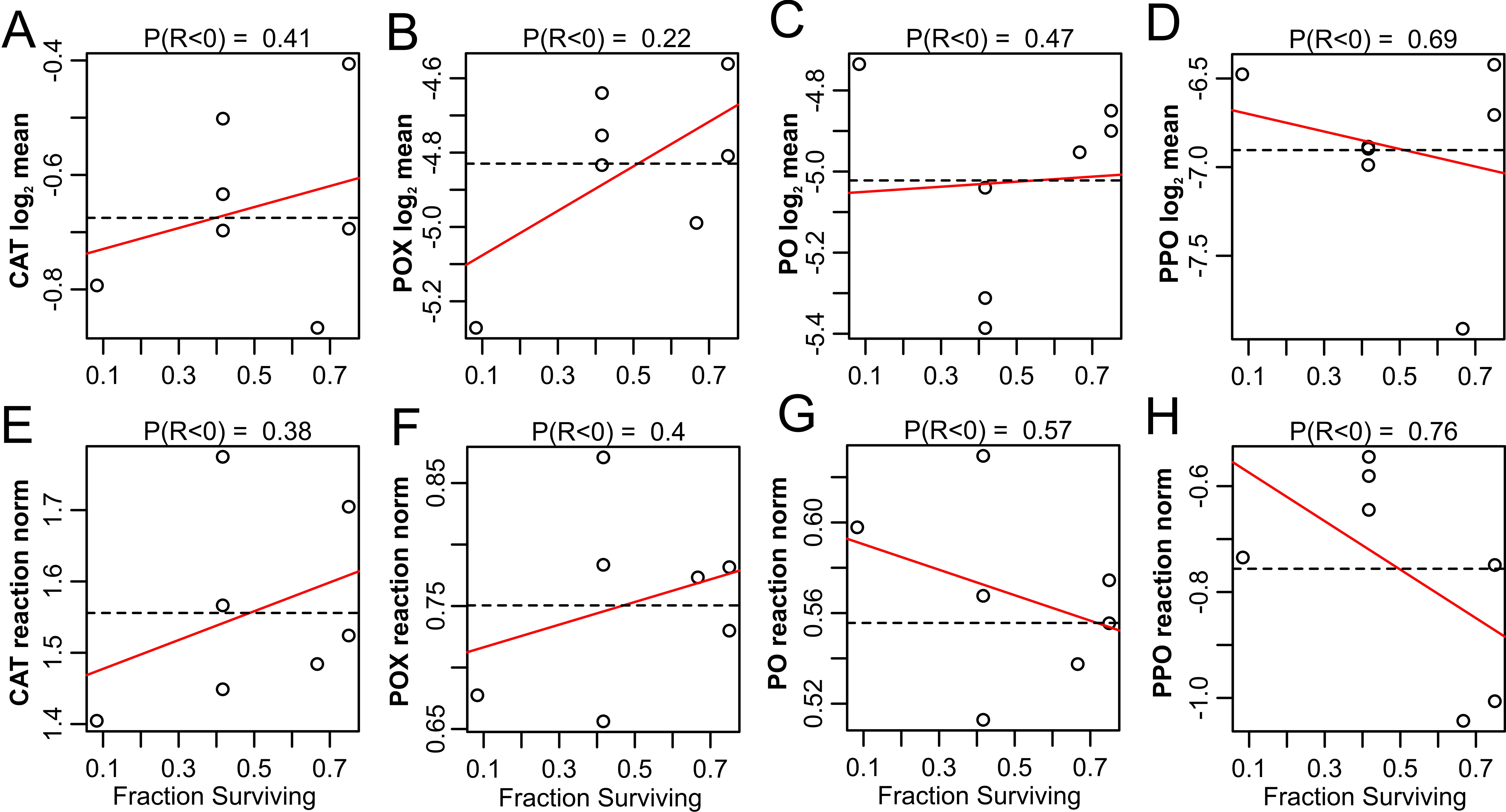


**Fig. S3: Correlations of mean immunity-related enzyme activities (A–D) and their reaction norms in response to bacterial challenge (E–H) with survival across genotypes, with genotype 30 excluded**. Mean catalase (CAT), peroxidase (POX), phenoloxidase (PO), and prophenoloxidase (PPO) activities are represented as log_2_-transformed Δ absorbance mg protein^-1^ min^-1^. Each point represents a posterior mean of the parameter for a genotype; dotted lines represent means across genotypes, red line is the linear model fit with survival as predictor variable. Value above the graph indicates posterior probability that the correlation with survival is negative.


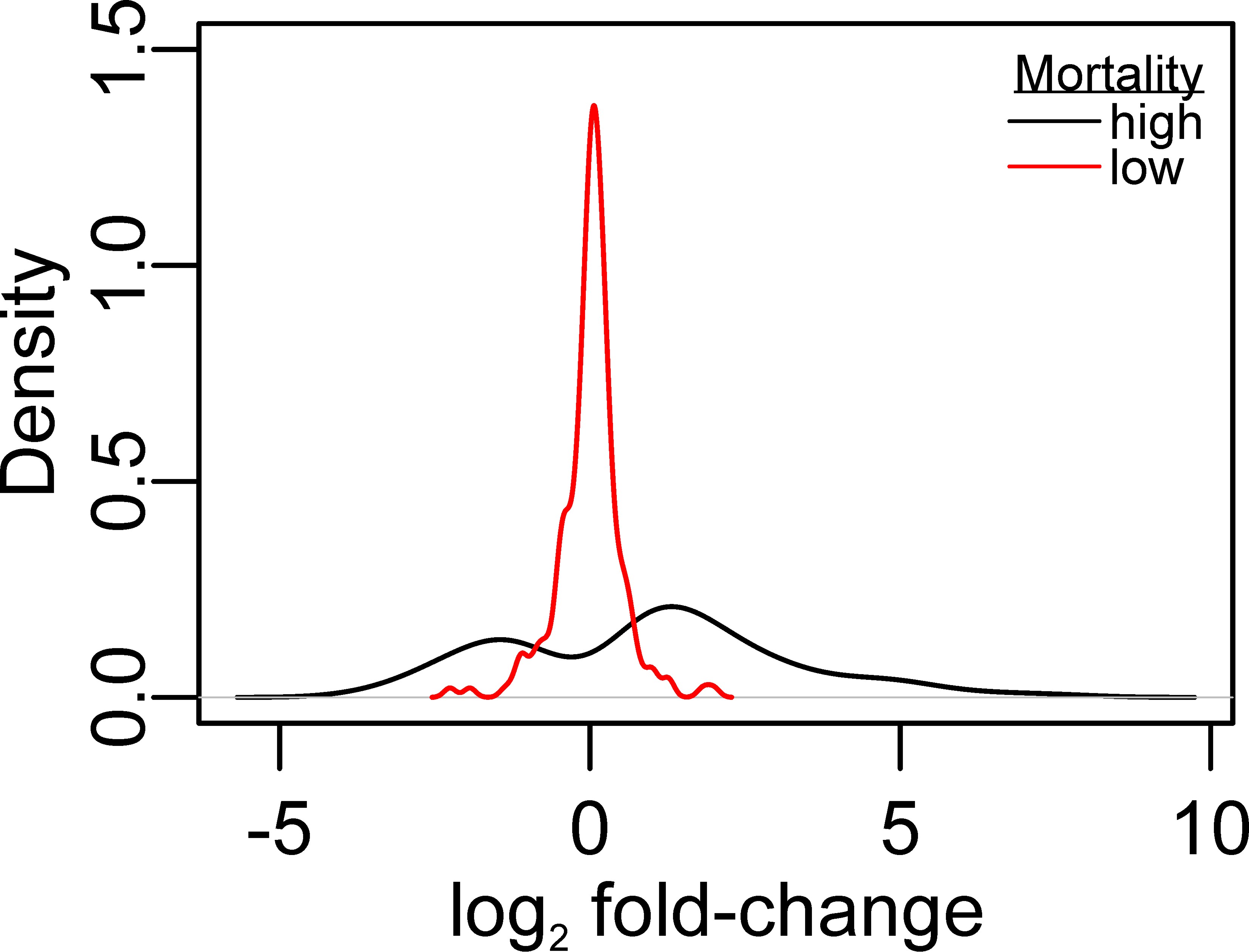


**Fig. S4: Density plots of predicted gene expression changes in response to bacteria treatment in the worst-surviving corals (survival = 0, black line) and best-surviving corals (survival = 1, red line).** Expression changes are extrapolated from the DESeq2 model incorporating bacterial challenge as a categorical predictor (yes or no), survival as a continuous predictor, and their interaction. The plots are based on 388 bacteria-responding DEGs identified at FDR = 0.1.

**
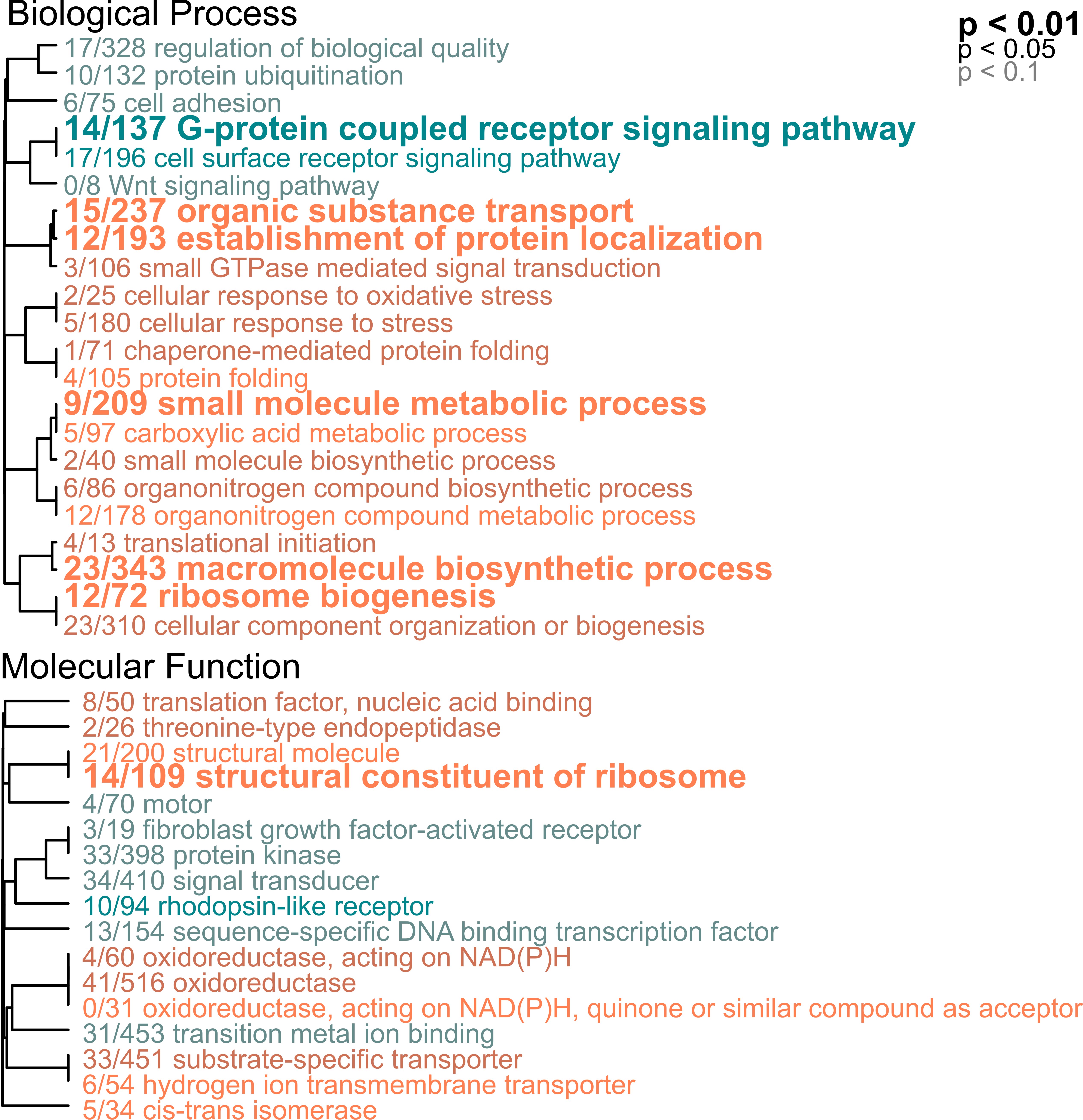
**

**Fig. S5: Gene ontology enrichment by interaction between treatment and survival.** Biological processes enriched according to the Wald statistic (log_2_ fold change divided by standard error) generated by testing the interaction between bacterial treatment (control and treated) and survival fraction (continuous). The text color indicates the direction of expression difference between corals with high and low survival (turquoise = more upregulated in lower survival corals, orange = more downregulated in lower survival corals). The text size indicates the significance of the term as indicated by the inset key. The fraction preceding the term indicates the number of genes within the term that had an absolute Wald statistic greater than 2. Trees indicate gene sharing among gene ontology categories (categories with no branch length between them are subsets of each other).


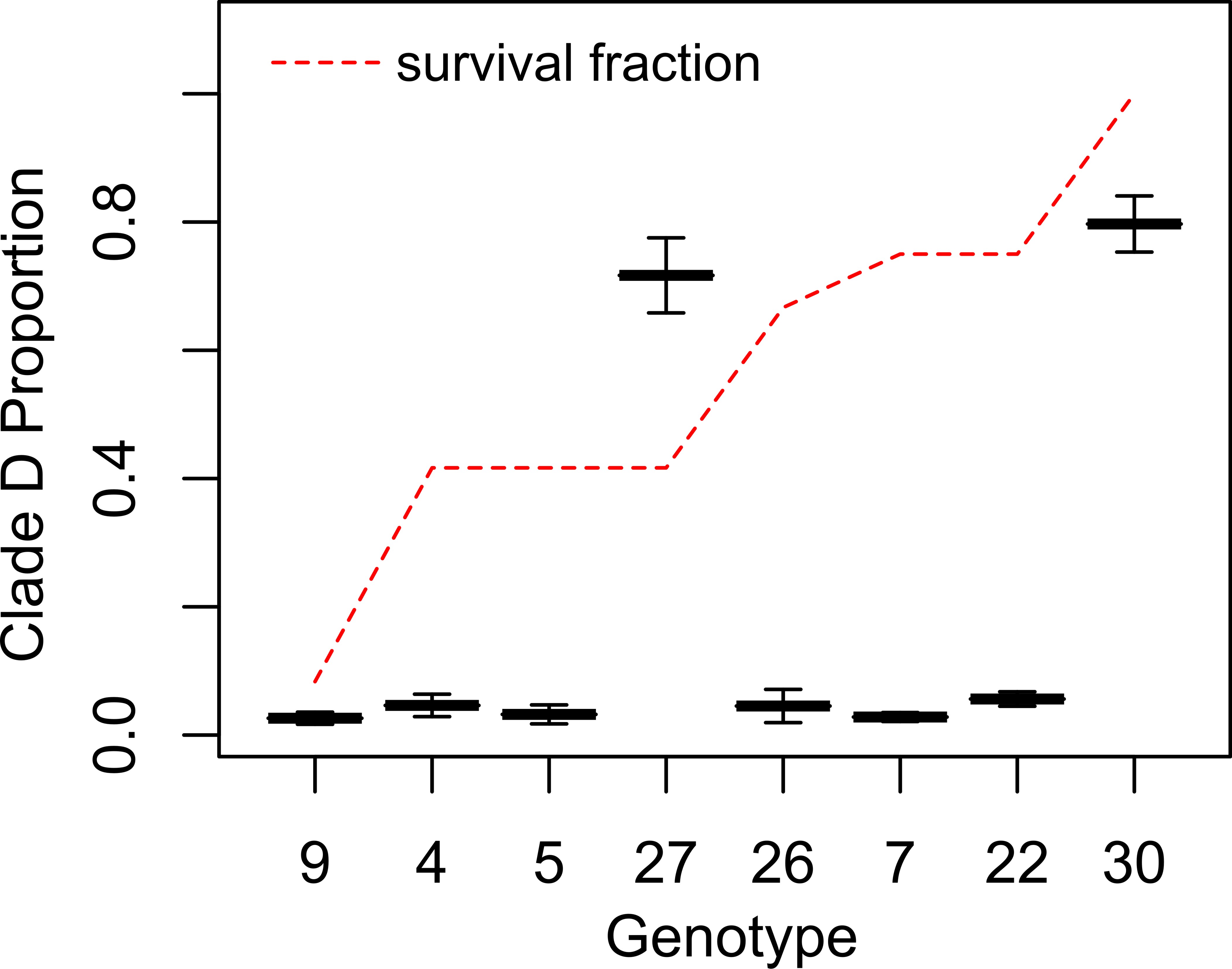


**Fig. S6: Proportions of clade D *Symbiodinium* in *A. millepora* based on RNA-seq reads mapping to clade D transcriptomes.** Genotypes 30 and 27 were dominated by clade D. All other genotypes were dominated by clade C. Genotypes are ordered by survival fraction increasing from left to right, which is indicated by the red dashed line. This is a boxplot of clade proportions in all TagSeq-analyzed fragments for a given genotype; the box (interquartile range) is not visible since the values are very similar for all fragments of the same genotype. The whiskers show 2.5X interquartile distance away from the median.





**Fig. S7: Example photos of each genotype and chloroplast-derived OTU counts. (A)** Genotypes are indicated in yellow text. Genotypes 4, 5, 7, 22, 27, and 30 were abraded with an airgun. Genotypes 9 and 26 demonstrate lesion development at ~50% tissue loss (time of death in this experiment). **(B)** Mean chloroplast-derived OTU counts (±SE) for each genotype normalized to total counts.


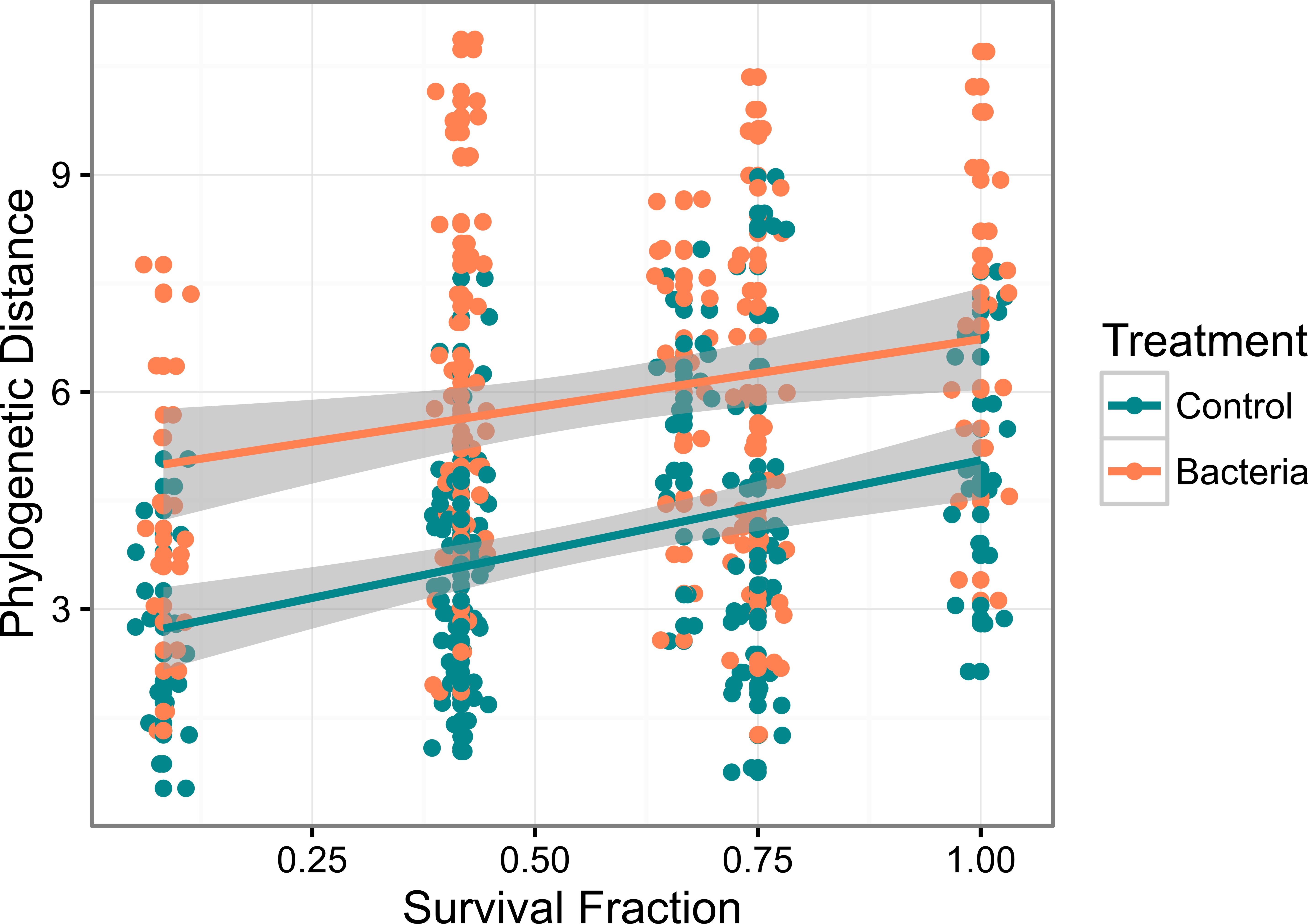


**Fig. S8: Phylogenetic diversity between treatments and mortality rates.** Faith’s phylogenetic diversity was calculated for each sample (n = 31) at ten sequencing depths ranging from 100 to 1000 reads. Each point represents a sample at a single sampling depth. Lines represent linear functions with 95% confidence regions shaded gray. Bacteria-challenged samples are orange, and controls are turquoise. Phylogenetic diversity of the microbial communities is higher in treated corals than controls (p < 0.001) and in corals with lower mortality (p = 0.04).

**
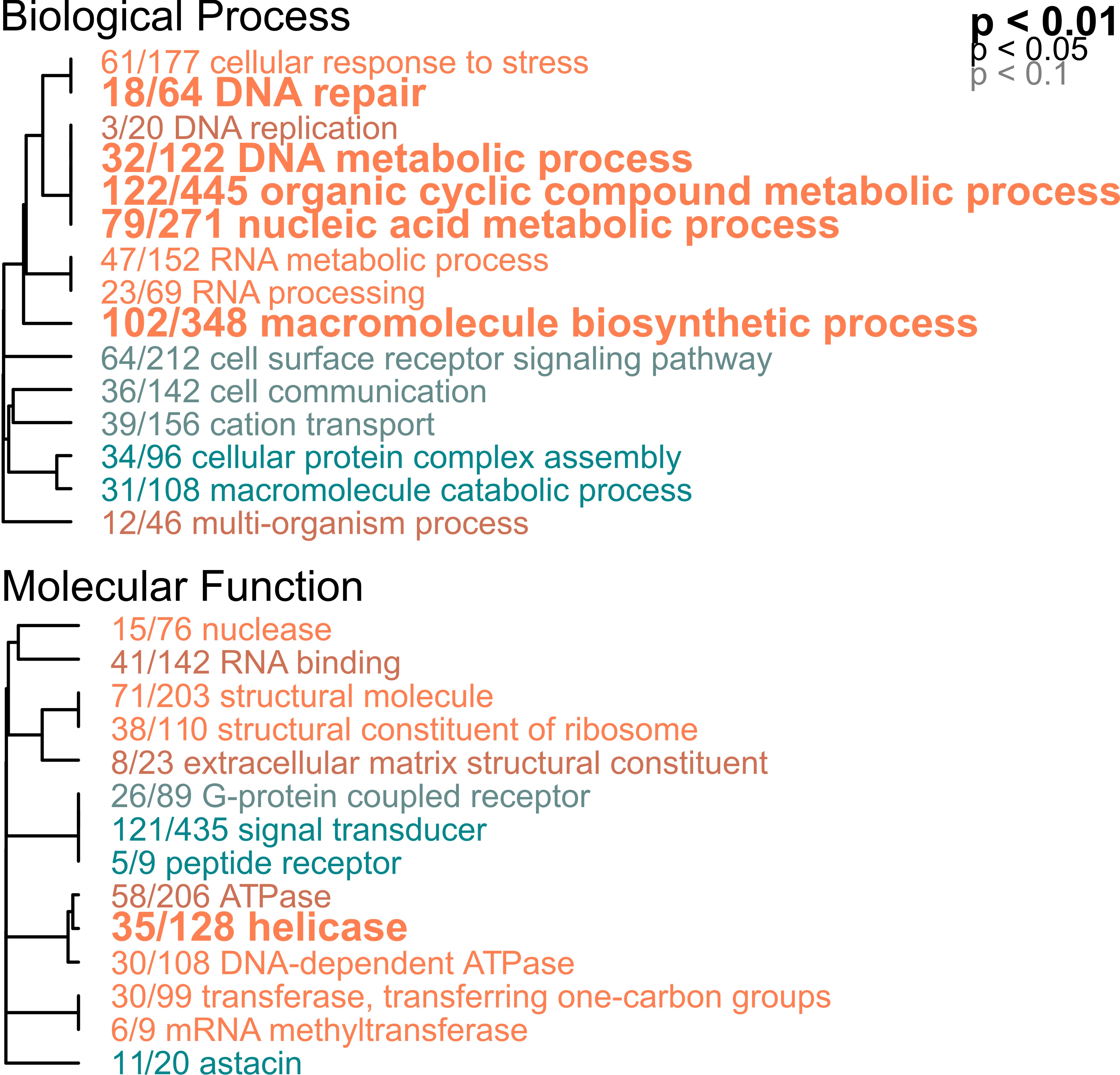
**

**Fig. S9: Gene ontology enrichment of genes differentially expressed by PCo1 value calculated using weighted UniFrac analysis.** Biological processes enriched according to the Wald statistic (log_2_ fold change divided by standard error) generated by modeling gene expression (n = 31) by PCo1 coordinate value (Fig. 5A). The text color indicates the direction of expression difference between corals with higher or lower PCo1 values (orange = upregulated in samples with higher PCo1 values; turquoise = upregulated in samples with lower PCo1 values). The text size indicates the significance of the term as indicated by the inset key. The fraction preceding the term indicates the number of genes within the term that had an absolute Wald statistic greater than 2. Trees indicate gene sharing among gene ontology categories (categories with no branch length between them are subsets of each other).

|  | **coefficient** | **exp(coefficient)** | **SE(coefficient)** | **z** | **p** |
| --- | --- | --- | --- | --- | --- |
| **L5** | -0.03 | 0.97 | 0.50 | -0.07 | 0.945 |
| **L7** | -1.61 | 0.20 | 0.69 | -2.35 | 0.0188 |
| **L9** | 1.08 | 2.96 | 0.47 | 2.32 | 0.0206 |
| **W22** | -1.58 | 0.21 | 0.68 | -2.31 | 0.021 |
| **W26** | -1.03 | 0.36 | 0.58 | -1.78 | 0.075 |
| **W27** | -0.25 | 0.78 | 0.50 | -0.49 | 0.6212 |
| **W30** | -19.40 | 0.00 | 4120.00 | 0 | 0.9962 |
| ***V. diazotrophicus*** | 1.74 | 5.67 | 0.43 | 4 | 6.20E-05 |
| ***V. owensii*** | 1.30 | 3.66 | 0.46 | 2.81 | 0.005 |
| **Abrasion** | 0.87 | 2.39 | 0.31 | 2.81 | 0.0049 |

**Supplementary Table 1: Stepwise Akaike information criterion analysis of Cox proportional hazards models to determine which factors in the experimental design affected mortality. The exponent of the coefficient is the ratio of the hazard rates of two levels of the explanatory variable. The hazard ratio for each genotype is expressed relative to L4. The hazard ratios for bacteria treatments (*V. diazotrophicus* and *V. owensii*) are expressed relative to the control condition. The abrasion effect is expressed relative to the non-abraded treatment.**

**Supplemental File 1: Perl script for quantifying Symbiodinium clades A, B, C, and D using RNA-seq reads.**

**Supplemental Data 1: DESeq2 results for all genes significantly differentially expressed (adjusted pval < 0.1) for the effect of bacterial treatment (Bac), survival (Surv), or the interaction of treatment and survival (Int).**

**Supplemental Data 2: OTU counts for all samples, including diseased sample “NO53” that was removed from the analysis.**

**Supplemental Data 3: DESeq2 results for all genes significantly differentially expressed by PCo1 value in the RNAseq dataset subset for 31 individuals with 16S data.**
